# Platinum and Rhodium in Potato Samples by Using Voltammetric Techniques

**DOI:** 10.3390/foods8020059

**Published:** 2019-02-05

**Authors:** Santino Orecchio, Diana Amorello

**Affiliations:** Chimiche e Farmaceutiche, Dipartimento di Scienze e Tecnologie Biologiche, Università di Palermo, Viale delle Scienze, 90128 Palermo, Italy; diana.amorello@unipa.it

**Keywords:** potatoes, platinum, rhodium, voltammetry

## Abstract

Potato is a starchy, tuberous crop from the perennial *Solanum tuberosum* having high nutritional values. This paper is the first analytical approach to quantify Pt and Rh in vegetal food. In this study a total of 38 different potato samples produced in Europe and one in Australia were investigated. Determinations of Pt and Rh in potato samples were carried out by Differential pulse voltammetry (DPV/a) for platinum and by Adsorptive stripping voltammetry (AdSV) for Rh using standard addition procedure. Because no certified reference potatoes containing platinum and rhodium are available, we used addition standard method. The quantification limits for Pt and Rh are 0.007 and 0.0008 μg kg^−1^ respectively. Considering all the potato samples, concentrations of Pt and Rh vary in the ranges from 0.007 to 109 μg kg^−1^ (sample no, 6 potatoes grown in Sicily) and from 0.0008 to 0.030 μg kg^−1^ (sample no. 3 of potatoes grown in Emilia Romagna), respectively. For both metals, in many cases the concentrations fall near the quantification limit. In all the samples, platinum is always more abundant than rhodium and their mean ratio is 14,500, which is much greater than that of the Earth’s crust (about 100).

## 1. Introduction

In most economically and socially evolved areas, the anthropic activity which appreciably has an effect on the environmental matrices quality is road transports [1,2]. Although, in latest years, traditional pollutant (NO, CO, hydrocarbons, lead, etc.) emissions decreased appreciably, the concentrations in the atmosphere of metals (Pt, Pd, Rh, Ru, and Ir), are increasing, in fact, these elements, known as platinum group elements (PGEs), are used in catalysts to reduce pollutants in the exhaust system.

Platinum group metals are principally emitted from vehicles in elemental form or as oxides. Because the low size of particulate in emitted gasses [3] and the several reactions which PGEs undergo, considerable amounts of these elements can be transformed into bioavailable compounds (chloro or organic complexes) [4]. Also Pt complexes are used as anticancer drug with high efficacy against solid tumors, particularly testicular and ovarian cancer [5,6,7]. Consequently, another source of this contaminant could be hospital incinerators. 

Soil is most susceptible environmental matrix to PGEs contamination [8,9]. Anthropogenic activity has caused an increase in PGE concentration in the soil, especially in areas close to roads, as proven by a number of studies [2,10]. Studies comparing the average concentration of Pt in different types of soil samples have shown that Pt concentration amounts to 0.14 µg/kg in intact soil (i.e., without human intervention) and 1.12 µg/kg in agricultural soil, while it reaches 20.9 µg/kg in soil specimens collected from areas adjacent to roads [11].

Few quantifications [12] of PGEs concentrations have ever been carried out in food from the time when these metals were used in car mufflers and as anticancer drugs and no information are available on platinum and rhodium concentrations in potatoes. 

Potato is a starchy, tuberous crop from the perennial *Solanum tuberosum*, introduced by the Spanish to Europe in the second half of the 16th century. Potatoes have high nutritional values, such as a high protein content (containing 18 essential amino acids, including various amino acids that the human body cannot synthesize), vitamins (including vitamin C and others that are useful for the human body), abundant dietary fiber, and little fat.

This paper is the first analytical approach to quantify Pt and Rh in vegetal food. In this study a total of 38 different potato samples produced in Europe and one in Australia were investigated.

Recently, a research group [13] investigated potentially toxic metals (As, Cd, Cr, Cu, Hg, Pb, Se, Zn), excluding PGEs, in agricultural samples (*Solanum tuberosum* L. tubers) in a zinc smelting area of Northwestern Guizhou Province (China). The health risks linked to potato consumption, respect to potentially toxic metal are a very important subject, because, today they are a primary food in many parts of the world. 

Potato quality control requires the development of reliable analytical methods to measure very low hazardous contaminant concentrations in food and environmental matrices, as well to estimate their background concentrations. In particular, the main intention of this work was to develop a reliable method for the quantification of Pt and Rh in complexes matrices, as potatoes, because they cannot be readily measured using conventional techniques employed in most laboratories, in particular, the Inductively coupled plasma emission spectrometry (ICP) techniques due to matrix and spectra interferences. Moreover, a direct determination of platinum and rhodium at ultra trace levels by Inductively coupled plasma mass emission spectrometry (ICP-MS) is difficult, due to the separation of this metal from the matrix and to interfering signals which cannot be eliminated [14,15,16,17].

In most cases, voltammetric methods are used for individual and simultaneous determination of metals and chemical compounds when their concentrations are very low (under ppb). Numerous applications of voltammetry are reported in literature [18,19,20] but to the best of our knowledge, there is no information on the determination of ultra traces of Pt and Rh [21,22,23,24] in a complex matrix as potatoes. In this research, adsorptive stripping voltammetry (AdSV) and the differential pulsed voltammetry (DPV) were used to measure the concentrations of Rh and Pt in the samples. 

## 2. Materials and Methods

### 2.1. Instrumentation

Instrumentation, laboratory apparatus, reagents, and procedures used were described in previous papers [8,9,25], however a brief summary is reported. The voltammetric instrumentation is constituted of a Polarograph Amel Model 433-A (Milano, Italy) with a glass cell, including a hanging mercury drop electrode (HMDE) (working electrode), a glassy carbon electrode (auxiliary), and an Ag/AgCl/KCl (sat) (reference electrode).

### 2.2. Reagents

The reagents used during this research were analytical grade (Carlo Erba, Milano, Italy) and all solutions were prepared in Milli-Q water. Platinum and rhodium standard solutions (1000 μg mL^−1^) were purchased from Fluka (Milano, Italy). The diluted solutions were prepared daily. HNO_3_ (65%) and HCl (37%) were analytical grade (Suprapur Carlo Erba, Milano, Italy). Hydrazine sulfate (N_2_H_6_SO_4_) and formaldehyde (HCHO) solutions were prepared daily from analytical-grade reagents (Carlo Erba, Milano, Italy). 

### 2.3. Quality Assurance

All the materials used during the analysis were cleaned before use by rinsing three times with HNO_3_ (3%) and three times with Milli-Q water. To avoid sample contaminations, different glassware and pipettes were used for standards and for solutions obtained from samples. The procedural blanks were routinely analyzed every six samples. Since certificate potatoes for the investigated platinum and rhodium are not available, all the analytical procedures were checked for accuracy by analyzing enriched samples prepared by us. The average recoveries of added analytes ranged from 80% to 95%. The relative standard deviations on the metals measurements of recovery are about 15%.

The detection (LOD) and quantification (LOQ) limits of the method, as in other studies [8,25], were calculated as the three- and ten-fold standard deviation of concentrations found in 10 procedural blanks, respectively, which were prepared in the same way as the potato samples.

### 2.4. Samples

The 38 analyzed samples had different geographical origins: Europe and Australia. Most samples were collected from Italy. Some of the samples are obtained from local farmers while others have been found on the market. In particular, 21 samples were potatoes from different parts of Sicily, 14 samples from northern Italy, two from France, and one from Australia. 

About 5–6 tubers of any type of sample were washed first with tap water and successively with Milli-Q water. The periderm was peeled immediately before analysis. Each tuber was cut into several similar small pieces using a ceramic knife. To obtain a representative sample, the tuber fragments were ground by a blender with a glass beaker and stainless steel blades.

### 2.5. Mineralization Procedure

A total of 2–3 g of finely ground sample, dried for 24 h at 105 °C, were ashed in a muffle at 600 °C (5 h). After cooling, the ashes were digested in 5 mL of concentrated HCl and filtered through 0.45 μm filters. After treatment was completed, the clear, colorless solution was transferred into a volumetric flask and brought to volume with Milli-Q water. The water content was determined by weight loss and was used to correlate all the results with dry weight.

### 2.6. Analytical Methods

Determinations of Pt and Rh in potato samples were carried out by Differential pulse voltammetry (DPV/a) for platinum and by Adsorptive stripping voltammetry (AdSV) for Rh using the standard addition procedure.

The solutions containing the complexes chloride of platinum (H_2_PtCl_4_, PtCl_4_^2−^) and rhodium (RhCl_3_, RhCl_6_^3−^) were purged with analytical-grade nitrogen (99.998%) at the start of each measure for 300 s and a flow of gas was maintained over the solution during the measure to prevent oxygen interference. All experiments were performed at a temperature of 25 °C.

Pt determinations were carried out in aqueous H_2_SO_4_ 1 M as the supporting electrolyte, in the presence of 1.2 mM hydrazine sulfate and 0.6 mM formaldehyde. Formaldehyde and hydrazine condense in situ to produce the corresponding hydrazone, which forms a complex with Pt. Subsequently, a potential varying from −0.3 to −1.0 V, in the differential pulse mode, was applied to the working electrode, and the catalytic current of the hydrogen formation was measured at −0.85 V (versus Ag/AgCl); its intensity being proportional to platinum concentration [17]. The catalytic effect of Pt makes this determination extremely sensitive.

Rhodium quantifications were carried out by adsorptive stripping voltammetry. This technique is known to give an incomparable sensitivity for several trace metals at a mercury electrode (film or drop); it involves complexation of metals with definite ligands and adsorption of the resulting complex on the mercury surface. The adsorbed complex is electrochemically removed by scanning the electrode potential, usually in a reductive direction. Since this is a surface technique, it is suitable for determining ultra-trace levels of metals in solutions.

In HCl (0.42 M) and HCHO (0.02 M) solution, a complex rhodium formaldehyde is adsorbed on a hanging mercury electrode at −0.7 V. The potential of the working electrode was then changed from −0.9 to −1.2 V, obtaining a peak at −1.1 V due to hydrogen reduction, catalyzed by rhodium complex. The catalytic effect of Rh explains the great sensitivity of the employed method.

**Figure 1:**
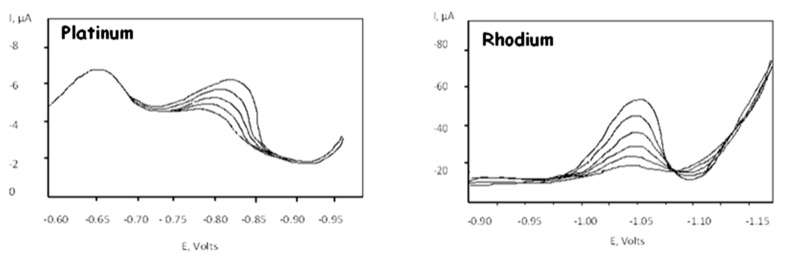
Voltammetric curves for platinum and rhodium.

**Figure 2:**
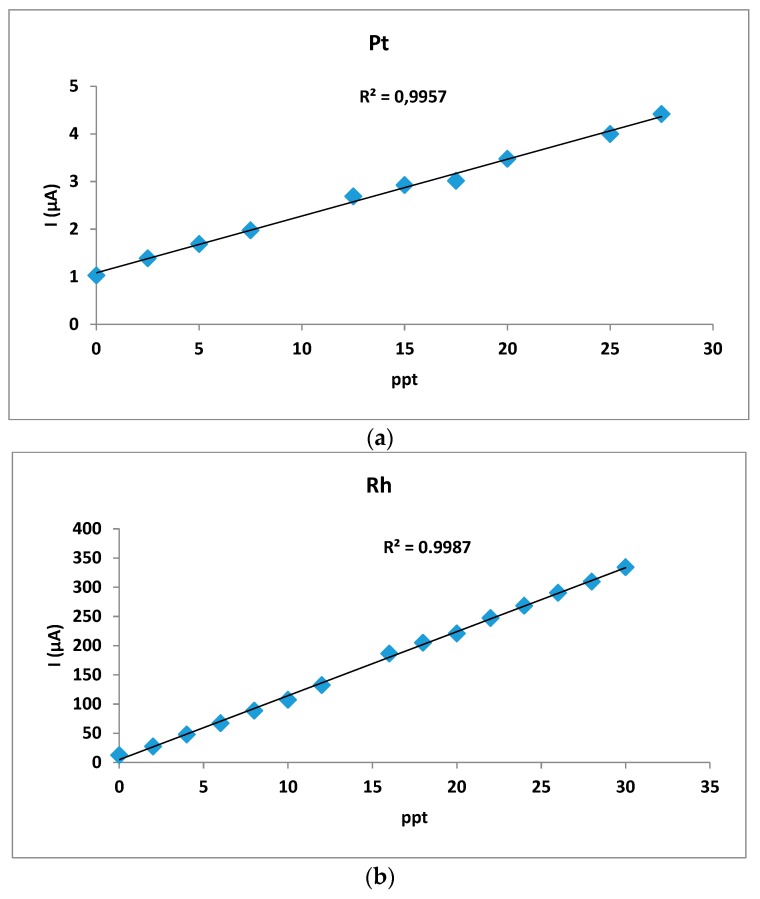
The calibration curves. (**a**) Voltammetric curves for platinum. (**b**) Voltammetric curves for rhodium.

The instrumental parameters are shown in Table 1 and Table 2. The voltammogram of backgrounds were obtained before sample analysis by using the same experimental conditions of the samples. Voltammetric curves for the two analytes are shown in Figure 1 and Figure 2. For both metals, quantitative measurements were performed using the standard addition procedure. Calibration graphs were built using data from measurements and evaluated by the least-squares linear regression method. Under the developed conditions, a very good linear correlation was obtained between the monitored voltammetric peak current and metals concentrations.

## 3. Results and Discussion

The narrow range of H_2_O (from 72 to 83%) found in the samples indicates, for this parameter, homogeneous chemical characteristics. 

Since no certified reference potatoes containing platinum and rhodium are available, in this study, we used addition standard method. This method was used to validate the analytical methods because sample matrix and possible interferences could also contribute to the analytical signal, a situation known as the matrix effect, thus making it impossible to compare the analytical signal (in our case current) between sample and standard using the traditional calibration curve approach. Using the intercept (negative) on the curve, the employed volumes of solutions and the quantity of samples, we have calculated the concentrations of Pt or Rh in the different potato samples. Calibration graphs were built using data from measurements and evaluated by the least squares linear regression method. Under the optimum conditions a very good linear correlations (R^2^ = 0.991–0.999 for platinum and 0.994–0.999 for rhodium) were obtained between the monitored voltammetric peak current and metal concentrations. The calibration curves, of which two (for platinum and rhodium) shown by way of example in Figure 2, indicate that the methods for the two metals are linear already starting from concentrations very close to the limits of quantification.

The precision of the developed methods, in terms of relative standard deviation (R.S.D. %) for Pt and Rh, ranged from 0.5 to 25 % and from 0.9 to 24%, respectively. Relative standard deviations are very high for those samples in which analyte concentrations are close to quantification limits.

The quantification limits for Pt and Rh calculated for the analyzed solutions obtained from the mineralization of the samples (in the voltammetric cell) are 13 and 1.6 ng L^−1^ respectively, while, considering the samples weight, the volume of analyzed solutions and the dilutions the quantification limits for Pt and Rh are 0.007 and 0.0008 μg kg^−1^, respectively. 

**Figure 3:**
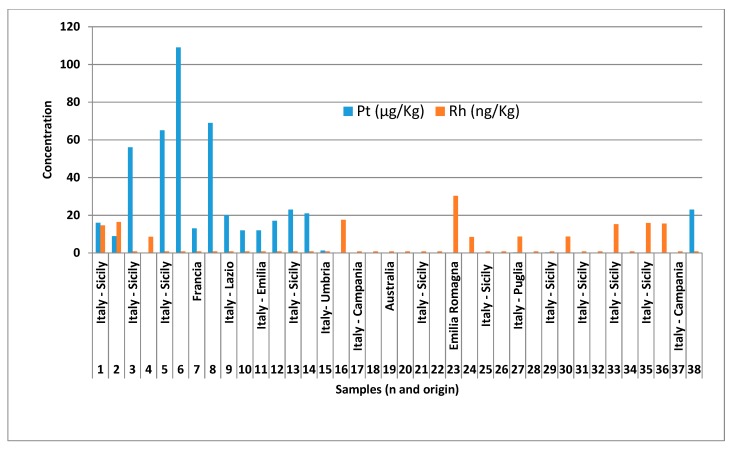
Platinum and rhodium concentrations in potato samples.

The concentrations of rhodium and platinum, obtained for the 38 analyzed potato samples are shown in Table 3 and Figure 3 and are referred to samples as such and not dried, exactly as they are consumed. 

The concentrations, reported as mean of three independent analyses, are corrected for blanks. Considering all the potato samples, concentrations of Pt and Rh vary in the ranges from 0.007 to 109 μg kg^−1^ (sample no. 6 potatoes grown in Sicily) and from 0.0008 to 0.030 μg kg^−1^ (sample no. 23 of potatoes grown in Emilia Romagna) respectively. For both metals, in many cases the concentrations fall near the quantification limit. In all the samples, platinum is always more abundant than rhodium and their ratio meanly is 14500, which is much greater than that of the Earth’s crust (about 100). 

The highest platinum concentrations were found in two samples cultivated in Sicily in the province of Syracuse, which is in a highly industrialized area due to the presence of refineries and other chemical industries.

We tried to treat the results from the statistical point of view but we realized that there is no correlation between the different samples nor between the platinum data between them nor between those of the rhodium, and even less between those of the two metals.

A comparison of the concentrations reported by us with those of literature is impossible due to the lack of data on the two analytes in the same matrix. For an evaluation, in Table 4 we show the platinum and rhodium concentrations determined by several researchers [26] in some plant and fungal species sampled along roads and motorways. In *Nerium oleander* leaves, Pt and Rh concentrations were found by us in the ranges 0.33–25 and 0.40–4.6 µg kg^−1^, respectively [25] (Table 4). These concentrations for platinum are of the same order of abundance than that observed in potato samples while Rh levels resulted lower.

Regarding platinum, in samples of German plants, the concentrations are meanly of the same size compared to that found in the potatoes analyzed by us (mean of 12 µg kg^−1^), while the concentrations of rhodium in the German samples are undoubtedly higher. 

A study on Pt concentration in the diet of Australian people was carried out about thirty years ago on market-basket samples [27]. Considering several food products from Sydney, the concentrations of Pt ranged from 8.1 μg kg^−1^ (liver sample) to 0.13 μg kg^−1^ (full-cream milk). In particular, Pt contents were highest (mean of 5.8 μg kg^−1^) in eggs and offal followed, in decreasing order, by meat (3.2 μg kg^−1^), grain products (3.2 μg kg^−1^), fish (1.8 μg kg^−1^), fruit and vegetables (0.82 μg kg^−1^), and products containing milk (0.27 μg kg^−1^) [27]. In Italian full cream milk, whole meal, and bread, the rhodium concentrations are 1.68, 0.14, and 2.2 μg kg^−1^, respectively [8].

The enrichment factor (EF) [28,29] can be used to differentiate between the contaminants originating from anthropic activities and those from natural processes and to assess the degree of anthropogenic influence.

EF, evaluated relative to the background values [30], was used to establish which elements were relatively enriched in the different samples. Values of EF close to 1 pointing to a natural origin while those >10 are considered to have a non-crustal source [28,29]. Further, EFs can also assist the determination of the degree of metal contamination. Five contamination categories are recognized on the basis of the enrichment factor (Table 5). In this study, considering Pt, the enrichment factors ranged from 6.5 × 10^−6^ to 011, whereas for the rhodium, the values of EF are in the range 0.08–0.30. The EFs calculated for all the samples indicate the natural origin of the two metals.

**Figure 4:**
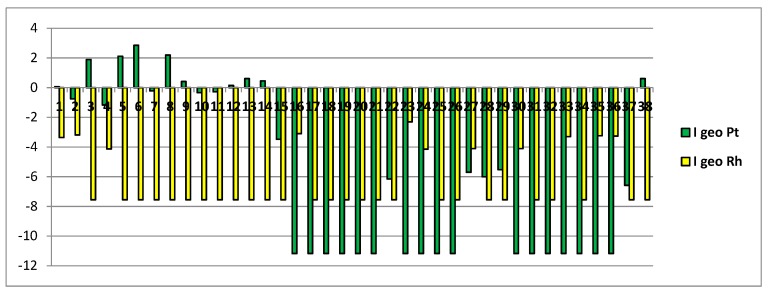
Geoaccumulation index values.

Additionally, in this paper, the degree of platinum and rhodium contamination in analyzed potato samples was characterized by the geoaccumulation index (Igeo) [29]:

I_geo_ = log_2_C_me_ = 1.5B_me_ (1)

where C_me_ is the measured concentration of metal in the sample and B_me_ is the geochemical background concentration in the hearth crust (Pt = 10 μg kg^−1^; Rh = 0.1 μg kg^−1^) [30]. The constant 1.5 allows us to consider natural fluctuations in the content of elements in the environment and to detect very small anthropogenic influences. Geoaccumulation index values for our data are shown in Figure 4.

For geoaccumulation index, different classes are given in literature [29,30] (Table 6). I_geo_ ranged from −11.2 to 2.9 with a mean of −0.27, and from −7.6 to −2.3 with a mean of −5.0 for platinum and rhodium, respectively. From the data results that, for platinum, about 80% of the samples could be classified as practically uncontaminated, five moderately contaminated, and three from moderately to heavily contaminated. The higher values of I_geo_ have been found in the potato samples (nos. 3, 5, 6, 8) grown in high-density industrial areas.

For rhodium, in all the cases, the I_geo_ indicates practically uncontaminated potato samples.

## 4. Daily Intake and Health Risk

Concerning the health risks derived from the intakes of platinum and rhodium eating potatoes, the results derived from this research are compared with the available toxicological values [31].

The Food and Nutrition Board, in agreement with what established by the Institute of Medicine (FNB), for the Pt, indicates a tolerable higher intake level of 0.3 μg per day per kg of body weight [31] in the adult which corresponds to about 15 μg per day for an individual weighing 50 kg [32]. The European Medicines Agency guideline recommends the permitted daily exposition (PDE) for Pt (100 μg d^−1^) and Rh (100 μg d^−1^) residues in drug substances. In the present paper, the PDE is assumed as the maximum acceptable exposure to Pt and Rh on a chronic basis that is unlikely to produce any adverse health effects.

The daily intake depends both on the level of metals in the food and the amount consumed. Daily intake (DIM) of metals was calculated using the following equation:

DIM = C_metal_ × D_food intake_ (2)

where C_metal_ and D_food intake_ represent the metal concentrations and daily intake of food, respectively. Considering that Americans, on average, eat 35 kilograms of frozen potatoes, 19 kg of fresh potatoes, 8 kg of potato chips and 6 kg of dehydrated potato products per year we considered 100 g/person/day [33]. Consuming the considered daily amount of potatoes, this supply from 0.0007 to 11 μg and from 0.00008 to 0.003 μg of platinum and rhodium for person, respectively. For comparison, the average diet of a Australian adult contains 1.4 μg of platinum per day (adult male, 1.7 μg Pt day^−1^; adult female, 1.2 μg Pt day^−1^), while in the United Kingdom the mean intake for rhodium is 0.2 μg day^−1^ [31].

## 5. Conclusion

In this study, the concentrations platinum and rhodium of great environmental and public interest in 38 different potato samples produced in several country were investigated. Only voltammetric techniques were used to quantify the two heavy metals. The advantages about the employ of these analytical techniques are the high sensitivity that improved the limits of quantification levels for the two elements that are presents at low levels in some samples, simplicity, speed and low costs. Analyzed potato samples contain concentrations of Pt and Rh under the recommended levels by international organisms for other food. In our case, for potatoes consumers the estimated intake of Pt and Rh through the studied common food was lower than the reported values. It is not to ignore the fact that, in Italy and in other European countries, many people consume daily amounts of this vegetable several times greater than those we have assumed.

## Figures and Tables

**Table 1:** Operating parameters for the voltammetric analysis (differential pulse voltammetry and adsorptive stripping voltammetry) of the solutions obtained from the ashes of potato samples.

Analytes	Tecniques	Electrolytes	Reagent
Pt	DPV/a	H_2_SO_4_ 1 M	(N_2_H_4_SO_4_) = 1.2 mmol L^−1^, (H_2_CO) = 0.6 mmol L^−1^
Rh	DPSAV	HCl 0.42 M	(H_2_CO) = 0.02 mol L^−1^

DPV/a: Differential pulsed voltammetry; DPSAV: Differential pulsed adsorptive stripping voltammetry.

**Table 2:** Operating parameters for the differential pulse voltammetry and adsorptive stripping voltammetry analysis of the solutions obtained from the potato samples.

Parameter	Pt	Rh
Initial potential (mV)	−300	−900
Final potential (mV)	−1000	−1200
Current range	Automatic	Automatic
Potential scan rate (mV s^−1^)	50	10
Potential of deposition (mV)	-	−700
Cycle n	1	1
Deposition time (s)	-	30
Stirring rate (r.p.m.)	300	300
Size of the drop (a.u.)	60	60
Delay time before potential sweep (s)	10	10
Working electrode	Hanging mercury drop electrode	
Auxiliary electrode	Glassy carbon	
Reference electrode	Ag/AgCl/KCl (sat)	
Flowing gas	Nitrogen (99.998%)	

**Table 3:** Platinum and rhodium concentrations in potato samples.

Sample	Origin	Pt (μg/kg)	R.S.D. % ±	Rh (μg/kg)	R.S.D. % ±
1	Italy - Sicily	16	2.2	0.015	5.4
2	Italy - Sicily	8.9	6.5	0.016	6.5
3	Italy - Sicily	56	2.4	0.0008	11
4	Italy - Sicily	6.7	6.1	0.0086	4.2
5	Italy - Sicily	65	1.0	0.0008	10
6	Italy - Sicily	109	0.5	0.0008	11
7	Francia	13	6.2	0.0008	20
8	Italy - Veneto	69	4.0	0.0008	12
9	Italy - Lazio	20	3.5	0.0008	9.9
10	Italy - Marche	12	5.2	0.0008	16
11	Italy - Emilia	12	4.7	0.0008	4.9
12	Italy - Emilia	17	2.3	0.0008	14
13	Italy - Sicily	23	2.4	0.0008	13
14	Francia	21	1.0	0.0008	13
15	Italy - Umbria	1.3	5.6	0.0008	5.6
16	Belgio	0.007	8.0	0.017	8.0
17	Italy - Campania	0.007	11	0.0008	21
18	Italy - Puglia	0.007	10	0.0008	25
19	Australia	0.007	12	0.0008	12
20	Italy - Abruzzo	0.007	16	0.0008	15
21	Italy - Sicily	0.007	12	0.0008	16
22	Italy - Sicily	0.21	3.2	0.0008	3.2
23	Emilia Romagna	0.007	12	0.0303	6.1
24	Italy - Sicily	0.007	13	0.0085	2.4
25	Italy - Sicily	0.007	11	0.0008	25
26	Italy - Sicily	0.007	18	0.0008	22
27	Italy - Puglia	0.288	12	0.0087	11
28	Italy - Abruzzo	0.233	15	0.0008	24
29	Italy - Sicily	0.326	15	0.0008	16
30	Italy - Sicily	0.007	10	0.0087	10
31	Italy - Sicily	0.007	17	0.0008	21
32	Italy - Sicily	0.007	14	0.0008	22
33	Italy - Sicily	0.007	15	0.015	0.88
34	Italy - Sicily	0.007	16	0.0008	24
35	Italy - Sicily	0.007	18	0.016	7.6
36	Italy - Sicily	0.007	25	0.016	7.2
37	Italy - Campania	0.16	14	0.0008	14
38	Italy - Sicily	23	1.2	0.0008	33

R.S.D.: Relative standard deviation.

**Table 4:** Platinum and rhodium concentrations in environmental matrices [26].

Place	Pt (μg/kg)	Rh (μg/kg)	Sample
Stuttgart	2.9 4.6	- -	Roadside grass (1993) Roadside grass (0.2 m) (1994)
Gent (Belgio)	1.4–1.7	-	Roadside grass
Germania	3.61	0.65	Roadside grass (1994)
	10.6	1.54	Roadside grass (1997)
	≤0.03	≤0.03	Area uncontaminated (1997)
Sheffield	0.07–5.4	-	Bark
Bialystok (Polonia)	8.63	0.65	Roadside grass (1 m)
San Francisco	38	-	Bark

**Table 5:** Contamination categories based on EF (enrichment factor) values.

EF < 2	Deficiency to Minimal Enrichment
EF 2–5	Moderate enrichment
EF 5–20	Significant enrichment
EF 20–40	Very high enrichment
EF N 40	Extremely high enrichment

EF: Enrichment factor.

**Table 6:** Geoaccumulation classes.

Class	Index	Significance
0	<0	Practically uncontaminated
1	0–1	Uncontaminated to moderately contaminated
2	1–2	Moderately contaminated
3	2–3	Moderately to heavily contaminated
4	3–4	Heavily contaminated
5	4–5	Heavily to extremely contaminated
6	5	Extremely contaminated
